# Factors influencing physical activity in postpartum women during the COVID-19 pandemic: a cross-sectional survey in Japan

**DOI:** 10.1186/s12905-022-01959-9

**Published:** 2022-09-08

**Authors:** Yumi Nomura, Tomoko Araki

**Affiliations:** 1 Faculty of Creative Engineering, Chiba Institute of Technology, 2-1-1, Shibazono, Narashino, Chiba 275-0023 Japan; 2 Department of Physical Therapy, Faculty of Health Science, Osaka Yukioka College of Health Science, 1-1-41, Sojiji, Ibaraki, Osaka 567-0801 Japan

**Keywords:** Physical activity, Postpartum, Anxiety, Quality of life, COVID-19

## Abstract

**Background:**

The aim of this study was to investigate factors influencing postpartum physical activity (PA), taking into consideration psychosocial perceptions during the coronavirus disease 2019 (COVID-19) pandemic by comparing health-related quality of life (HRQoL) scores.

**Methods:**

A web-based cross-sectional survey of 787 postpartum women was conducted between March and October 2021. After applying the exclusion criteria, 590 women were analyzed. The International Physical Activity Questionnaire Short Form, was used to assess the level and amount of PA. The Short Form-12 Health Survey version 2 (SF-12v2) was used to measure HRQoL. Logistic regression analyses were used to determine whether sociodemographic factors and psychosocial perceptions during the COVID-19 pandemic were associated with the level of PA. Based on the current national guidelines for exercise in Japan, respondents were classified by weekly PA level as an Inactive group and an Active group to assess the influence of PA on HRQoL.

**Results:**

Mean total PA was 19.3 total metabolic equivalents hour/week, and the prevalence of an inactive lifestyle was 45.9% among respondents. Each year of age was associated with an odds ratio (OR) of 0.92 (95% CI 0.87–0.97) for becoming physical inactivity during postpartum. Factors positively associated with more active levels were greater number of days for delivery (OR = 1.00; 95% CI 1.00–1.01), multiparity (OR = 1.50; 95% CI 1.00–2.23), having someone to talk about childcare and the individual’s partner (OR = 2.04; 95% CI 0.96–4.36) and not having anxiety symptoms (OR = 0.58; 95% CI 0.35–0.97). The Active group had significantly higher HRQoL scores than the Inactive group in the following scales: physical component summary (*p* < 0.001), mental component summary (*p* = 0.041).

**Conclusions:**

The influential factors for postpartum PA level were younger age, longer duration after childbirth, multiparity and not having anxiety symptoms, which correlated positively with PA. The presence of someone with whom can talk to about childcare and partner issues was associated with the maintenance of higher PA among postpartum women, suggesting that factor as a positive influence on PA under unsettled conditions.

## Background

Physical activity (PA) is well established among the maternal population as having a protective effect on both mental and physical health such as reduction in depression and anxiety. On 11 March 2020, the World Health Organization (WHO) declared the novel coronavirus disease (COVID-19) outbreak a global pandemic, resulting in significant restrictions on movement in the daily life globally. The Japanese government did not mandate a lockdown, but ask ordinary people to stay at home and keep social distancing by self-refrain (this was called “Jisyuku” in Japanese). Although the Japanese government’s policy had no legal effect, the number of steps per day decreased by 15% from the pre-declaration level for 24 days after the pandemic declaration (during first self-refrain requests) [1]. One of the most vulnerable populations is postpartum women, since most support systems and services were canceled at birth facilities and public health centers under the new infection control measures. Several studies have indicated a number of deleterious consequences of isolation among pregnant and postpartum women, including increases in depression and anxiety [2], and reduced PA [3]. The prevalence of postpartum depression in Japan was 11.5–16.3% in a meta-analysis covering 1994–2017 [4], and 28.7% in a survey conducted in October 2020, after the pandemic was declared [5]. A recent study also indicated that maternal women who met the guidelines of at least 150 min/week of moderately intense PA during the pandemic were significantly less depressed and anxious than women who did not [3].

Therefore, we made a hypothesis that self-refrain requests might decrease PA among postpartum women, which might lead to a decline in their health status. Moreover, pregnancy and postpartum represent critical periods associated with significant physical and psycho-social changes that usually result in decreased PA [6]. Recent reports have reported subjective changes in postpartum PA before and after the pandemic [3, 7], but quantitative measurements of PA (e.g., energy consumption and activity level) and related factors during the COVID-19 pandemic have not been elucidated. Factors related to PA under this unsettled emergency, thus need to be investigated and measures to promote PA considered. To clarify the impact of the COVID-19 pandemic, interactions between individual, interpersonal and environmental factors need to be explored, consistent with a socioecological approach [8]. During the postpartum period, individual factors could include general and mental health, and lifestyle behaviors; interpersonal factors could include working and income status, and childcare support; and environmental factors could include the availability of the resources needed to exercise [9–11]. However, these factors, particularly mental health and childcare support have changed due to the COVID-19 pandemic and may also affect PA. Actually, postpartum women who experienced COVID-19-related social restrictions (e.g., loss of support from parents or other family members, loss of opportunities to consult with friends about childcare) were twice as likely to experience postpartum depression [5].

The aim of this study was to investigate factors influencing postpartum PA, taking into consideration the psychosocial perceptions during the COVID-19 pandemic by comparing the health-related QoL (HRQoL) scores of postpartum women in Japan.

## Methods

### Ethics approval

This study was approved by the human research ethics committee of Nippon Sport Science University, and is consistent with their requirements for human experimentation (approval no. 020-H149). All procedures performed in studies involving human participants were undertaken in accordance with the Declaration of Helsinki and the ethical standards of the institutional and national research committee. A check box for informed consent was provided before proceeding to the response page of the online questionnaire form.

### Study design and participants

**Fig. 1:**
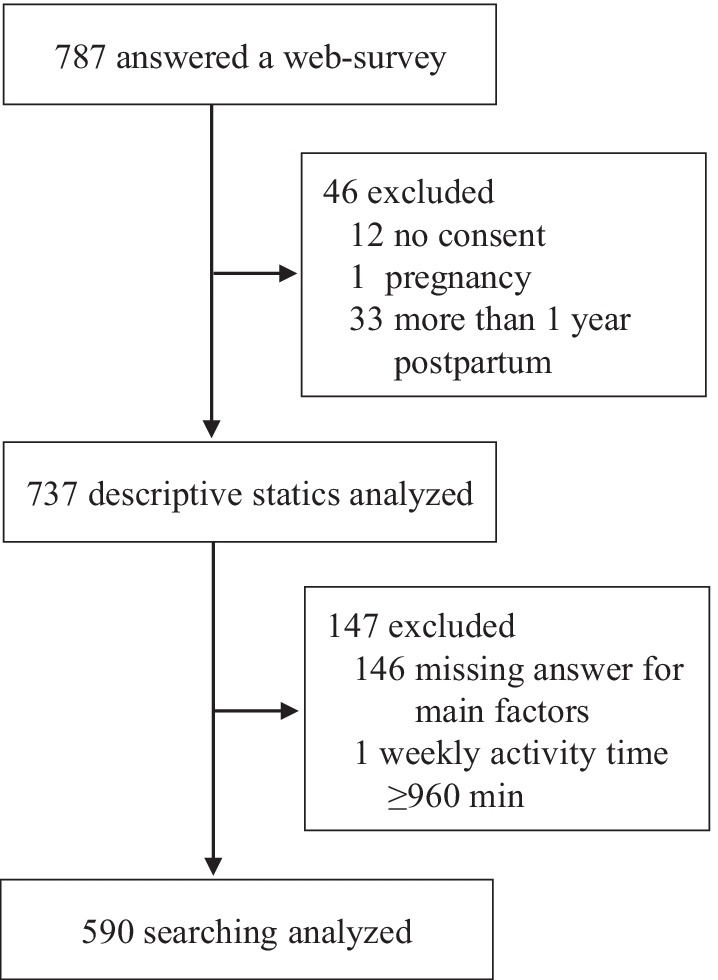
Flowchart of participant selection for the study. ICF, informed consent form

This research was conducted as part of the study mental and physical status, and the partnership of pre/postpartum women in COVID-19 pandemic, a cross-sectional study in Japan. To prevent the spread of COVID-19 through contact, data were collected using a web-based survey. Between March and October 2021, we recruited postpartum women who had given birth after March 2020 (declaration of the COVID-19 pandemic) to participate in a web-based survey. Eligible women were: (1) over 20 years old; (2) living in Japan; (3) without any medical complications; and (4) within the first year after delivery or expecting to give birth after March 2020. The following exclusion criteria were applied: pregnant women (n = 1); consent not entered or declined (n = 12); or missing data from the questionnaire about PA and HRQoL (n = 179). In accordance with the International Physical Activity Questionnaire Short Form (IPAQ-SF) guidelines, we excluded one case with a total weekly activity time ≥ 960 min (Fig. 1). A final total of 590 postpartum women was included in the analysis.

### Data collection

The research guidelines and application were posted to the homepage of a Japanese non-profit organization that provides healthcare information and programs for postpartum women (Madrebonita, Tokyo, Japan) and shared publicly. After receiving an application, the research team sent the prospective participant a personally addressed e-mail with a hyperlink to the questionnaire on the Google platform. In addition, the e-mail explained that the data obtained would be confidential and that the research team was independent of the organization and any other organization.

### Questionnaires

The web-based survey comprised 37 questions categorized into 5 main sections: (1) socio-demographic/maternity-related information (Q1–Q8); (2) psychosocial perceptions during the COVID-19 pandemic, in terms of childcare support (Q9, Q10), change in economic status (Q11), increased burdens of housework and childcare (Q12), behavioral restrictions due to the COVID-19 pandemic (Q13), relationship with a partner (Q14); (3), the Whooley Questions [12] as a screening tool for depression (Q15, Q16), and the two-item Generalized Anxiety Disorder scale (GAD-2) [13] as a screening tool for anxiety (Q17, Q18); (4) SF-12v2 as a measure of HRQoL (Q19–30); and (5) PA (Q31–37).

### Dependent variable

#### PA

IPAQ-SF was used to assess the level and amount of PA achieved by respondents [14]. The IPAQ-SF contains questions about the amounts of “walking”, “moderate physical activity”, and “vigorous physical activity” within the last 7 days. Participants were asked about the specific number of days and the amount of time in minutes spent doing these respective activities performed for at least 10 consecutive minutes each day. Total, vigorous, moderate, and walking metabolic equivalents (MET)-hours per week were calculated according to the IPAQ scoring protocol [15]. Three levels of PA have been proposed by the IPAQ group, as follows: inactive; minimally active; and more active. The Japanese Ministry of Health, Labour and Welfare recommends that adults between 18 and 64 years old engage in 23 MET hours per week of PA to promote and maintain health [16]. These values correspond to the ‘minimally active’ category of the IPAQ-SF. Moreover, a previous study has indicated that fewer subjects categorized in ‘more active’ of the IPAQ-SF in Japanese postpartum women [17]. Thus, for the purposes of this study, we reclassified IPAQ categories into two groups: inactive as the ‘Inactive group’; and minimally or more active as the ‘Active group’.

### Independent variables

#### Depressive and anxiety symptoms

To examine the current status of depression and anxiety, we used two screening tools recommended by the National Institute of Health and Care Excellence guidance [7]. The first tool was the Whooley Questions [12], a screening instrument for depression in the general adult population, including maternal women: “Have you often been bothered by feeling down, depressed or hopeless?” (yes/no); and “Have you often been bothered by having little interest or pleasure in doing things?” (yes/no). If a “yes” response was obtained for at least one of the two questions, the person was defined as having depressive symptoms (0: negative; 1: positive). The second tool was the GAD-2　to screen for generalized anxiety disorder [13]. These questions were: “Have you been bothered by feeling nervous, anxious almost every day?” (yes/no); and “Have you been bothered by not being able to stop or control worrying almost every day?” (yes/no). If the answer to at least one of the two questions was “yes”, that person was defined as having anxiety symptoms (0: negative; 1: positive). These screening tools for depressive and anxiety symptoms are reportedly useful for detecting women at high risk of postpartum depression [13, 18].

#### Psychosocial perceptions during the COVID-19 pandemic

A previous study has indicated that perceived barriers such as more responses for childcare [19], lack of childcare [9, 20], and support by partner [19] may affect postpartum PA. Nagata et al. revealed that decreased walking activity and increased sedentary behavior were associated with socioeconomic status and anxiety related to going out during the first wave of the COVID-19 pandemic in Japan [21]. Based on these previous studies, we asked specific COVID-19-related questions in relation to the impact of COVID-19 on the lives and relationships of the respondent, as follows: number of people participating in childcare other than the respondent (Q9); person to whom the respondent can talk about childcare and partner (0: no; 1: yes) (Q10), negative change in economic status (0: yes; 1: no) (Q11), increased role for housework and childcare (0: yes; 1: no) (Q12), and behavioral restrictions related to the pandemic (0: yes; 1: no) (Q13).

#### HRQoL

SF-12v2 is widely used as a standard measurement tool for HRQoL using a self-administered questionnaire [22]. The SF-12v2 is a shortened version of the Short Form-36 Health Survey (SF-36). The SF-36 and SF-12v2 are the two most frequently used measures of HRQoL for pregnant and postpartum conditions [23]. Both SF-36 and SF-12v2 have previously been translated into Japanese and offer confirmed validity and reliability in Japanese populations [24]. Items of the SF-12v2 are summarized into two weighted scales (Physical Component Summary scale, PCS; Mental Component Summary scale, MCS) designed to assess physical and mental well-being. Each is scored to have a mean of 50 and standard deviation (SD) of 10 in the Japanese population, with lower scores indicating higher levels of impairment.

### Statistical analysis

Total weekly PA was computed by summing of total MET hour/week (h/wk) in walking, moderate-intensity activity, and vigorous-intensity activity. To test the first objective, participants were classified by PA level (Inactive group / Active group) to investigate factors of PA using logistic regression analyses. Logistic regression models estimated the likelihood of being in the Inactive group relative to the Active group a function of predictor variables: sociodemographic factors and psychosocial perceptions during the COVID-19 pandemic. To test the second objective, Mann–Whitney *U* test was used to compare the HRQoL scores between Inactive group and Active group. Statistical assumptions associated with linear regressions (normality of residuals, homogeneity of variance, linear, multicollinearity, and undue influence) were checked and models were adjusted as required (e.g., outlier removal). Statistical analysis was performed using IBM SPSS Statistics version 28 (IBM SPSS Japan, Tokyo, Japan). Values of *P* < 0.05 were considered to indicate statistical significance for all analyses.

## Results

### Sample characteristics and descriptive statistics

Table 1 shows the characteristics of the participants. The mean age of participants was 34.9 years, and mean period after delivery was 135.4 days. Proportions of participants who > 35 years old (late childbearing), primipara, delivered vaginally, had a nuclear family, had high educational attainment (> 13 years) and were employed workers were 54.1%, 44.7%, 74.7%, 95.4%, 92.0% and 83.7%, respectively. Exercise habits during school days were reported by 34.7% of women. Participants were categorized into an Inactive group (45.9%) and an Active group (54.1%).

**Table 1:** Participant characteristics and descriptive statics

	Mean (SD)	n (%)
Age, year	34.9 (4.0)	
BMI, kg/m^2^	21.3 (2.4)	
Days from the date of birth	135.4 (76.0)	
*Parity*		
Primipara		264 (44.7)
Multiparous		326 (55.3)
*Education*		
< 13 years		41 (6.9)
≥ 13 years		543 (92.0)
Missing		1 (0.0)
*Working status*		
Non-worker/student		94 (15.9)
Worker		494 (83.7)
Missing		2 (0.3)
*Psychosocial perceptions during the pandemic*		
Number of members who have been involved in childcare on a daily basis	1.3 (0.7)	
*Person who can talk about childcare and partner*		
Yes		479 (81.2)
No		35 (5.9)
Missing		76 (0.1)
*Negative change of economic status*		
Yes		67 (11.4)
No		520 (88.1)
Missing		3 (0.5)
*Increased role of housework and childcare*		
Yes		123 (20.8)
No		467 (79.2)
*Behavioral restrictions related to the pandemic*		
Yes		511 (86.6)
No		77 (13.1)
Missing		2 (0.3)
*Screening for depressive and anxiety symptoms*		
Depression		
Positive		183 (31.0)
Negative		330 (55.9)
Missing		77 (13.1)
Anxiety		
Positive		96 (16.3)
Negative		418 (70.8)
Missing		76 (12.9)

SD, standard deviation; BMI, body mass index

Median with interquartile range (IQR) and levels of PA are shown in Table 2. Median MET values for total, vigorous, moderate, and walking activity were 13.2 h/wk (IQR 6.6–23.2), 0.0 h/wk (IQR 0.0–0.0), 0.0 h/wk (IQR 0.0–13.2), and 9.9 h/wk (IQR 5.0–19.8). The prevalence of an inactive lifestyle was 45.9% among postpartum women.

**Table 2:** Median (IQR) and level of self-reported physical activity

	Median (IQR)	n (%)
*Physical activity (MET h/wk)*		
Vigorous	0.0 (0.0–0.0)	
Moderate	0.0 (0.0–13.2)	
Walking	9.9 (5.0–19.8)	
Total	13.2 (6.6–23.2)	
*Physical activity level*		
Active (more and minimally)		319 (54.1)
More		31 (5.3)
Minimally		288 (48.8)
Inactive		271 (45.9)

MET h/week, metabolic equivalents hours per week; IQR, interquartile range

### Logistic regression analyses

Logistic regression results for odds ratio (OR) of active lifestyle according to women characteristics are shown in Table 3. The most important explanatory factor on the PA was each year of age (OR, 0.92; 95% CI 0.87–0.97), which was negatively associated with PA. Each day for delivery was associated with an OR of 1.00 (OR = 1.00; 95% CI 1.00–1.01) for becoming higher PA level. In comparison to primiparous women, multiparous women appeared to be higher PA level (OR = 1.50; 95% CI 1.00–2.23). Women having anxiety symptoms had an OR of 0.58 (OR = 0.58; 95% CI 0.35–0.97) for becoming low PA level. Additionally, the presence of someone to talk to about childcare and partner during the COVID-19 pandemic (OR = 2.04; 95% CI 0.96–4.36) was positively associated with PA.

**Table 3:** Logistic regression results of sociodemographic / COVID-19 related factors to active lifestyle

Variables mean (SD) or %	Inactive group n = 271	Active group n = 319	OR	95% CI	*P* value
Age, year	36.2 (3.4)	34.5 (4.1)	0.92	0.87–0.97	0.002
BMI, kg/m^2^	22.1 (2.6)	21.1 (3.0)	0.97	0.90–1.05	0.451
Days from the date of birth	137.3 (84.5)	154.5 (81.0)	1	1.00–1.01	0.024
Multiparity (ref: primiparity)	54.2	56.1	1.5	1.00–2.23	0.049
Worker (ref: non-worker)	5.2	5.7	0.98	0.43–2.25	0.962
*Psychosocial perceptions during the pandemic*					
Number of members who have been involved in childcare on a daily basis	1.2 (0.5)	1.3 (0.6)	1.16	0.70–1.93	0.564
Having someone to talk about childcare and partner	90.5	95.4	2.04	0.96–4.36	0.065
Negative change of economic status	10.4	12.2	1.07	0.61–1.87	0.805
Increased role of housework and childcare	22.1	19.7	1.15	0.74–1.80	0.524
*Screening for depressive and anxiety symptoms*					
Depression (ref: negative)	36.2	35.2	1.1	0.72–1.68	0.669
Anxiety (ref: negative)	22	16	0.58	0.35–0.97	0.039

OR, odds ratio; CI, confidence interval

### PA level and HRQoL

Differences in SF-12v2 between inactive and active levels are shown in Table 4. The Active group showed significantly higher the HRQoL scores than the Inactive group in the following SF-12v2 scales: physical component summary (*p* < 0.001), mental component summary (*p* = 0.041), physical function (*p* < 0.001), role (physical) (*p* < 0.001), bodily pain (*p* = 0.001), general health perceptions (*p* < 0.001), vitality (*p* < 0.001), role (emotional) (*p* = 0.005), and mental health (*p* = 0.024). No significant differences in social functioning were evident between groups (*p* = 0.107).

**Table 4:** Health-related quality of life between those who were inactive and active levels of physical activity

	Inactive group	Active group	*P* value
*SF-12v2 component summaries*			
Physical component summary	47.3 (10.4)	50.5 (8.4)	< 0.001
Mental component summary	54.5 (7.3)	55.8 (7.5)	0.041
*SF-12v2 sub-scales*			
Physical function	49.7 (9.7)	52.4 (7.1)	< 0.001
Role (physical)	37.3 (10.9)	41.7 (10.5)	< 0.001
Bodily pain	43.3 (11.0)	46.3 (10.3)	0.001
Social functioning	44.4 (11.4)	46.0 (11.9)	0.107
General health perceptions	50.2 (8.0)	52.6 (7.9)	< 0.001
Vitality	52.4 (7.3)	55.3 (7.5)	< 0.001
Role (emotional)	41.7 (10.8)	44.1 (10.2)	0.005
Mental health	49.7 (7.8)	51.2 (7.8)	0.024

Values are means (SD)

## Discussion

The three main findings of this study are summarized as follows: First, the half of the postpartum women showed inactive levels of PA during the self-refrain situation in Japan. Second, the influential factors for higher PA level were younger age, longer duration after childbirth, multiparity and not having anxiety symptoms, which correlated positively with PA. Third, mothers with active PA level had higher HRQoL scores than mothers with inactive PA level.

### Postpartum PA under self-refrain requests in Japan

The mean of total PA of participants was 19.3 MET h/wk, and the percentage of adults aged 18–64 years who achieved the recommended PA of 23 MET h/wk as advocated by the Japanese Ministry of Health, Labor and Welfare was 28.6%. Reportedly, 17.2% of Japanese women in their 30 s achieved the recommended PA, lower than in other generations [25], and women in the child-rearing generation tend to show lower PA. In this study, the percentage of low activity level in the IPAQ was 45.9%, indicating that participants in this study were more active compared to a previous study of Japanese postpartum women (92.0%) [17]. Increased sedentary time and screen time and physical inactivity during the COVID-19 pandemic represent contributors to worsen mental health [8], suggesting that early detection through screening and intervention are needed.

### Factors influencing PA in postpartum women

This study investigated the relationship between postpartum PA and psychosocial aspects during self-refrain requests in Japan. The results showed that longer duration after childbirth and multiparity were shown to have positive effects on PA levels. A new finding is that anxiety symptoms, as screened by GAD-2, have a negative impact on among Japanese postpartum women during the government's request for self-restrain. Moreover, women who have someone with whom they can talk to about work and family life had a higher total PA. From the time the pandemic was declared in March 2020, activities of daily living were restricted at social and individual levels, representing a source of mental health problems [2, 3, 5]. According to a systematic review of pregnant and postpartum women, the prevalence of mental health disorders after the pandemic started was higher than before the pandemic for both anxiety (40%) and depression (27%), with high prevalence of anxiety both during pregnancy and in the postpartum period [2].

In the time after childbirth, total PA at 12 months postpartum reportedly increased by 1 MET h/wk compared with 3 months postpartum, with a higher percentage of PA from going out in association with childcare and family activities [6]. Opportunities to go out the increase as the child grows, which is conjectured to increase PA. In addition, the depressive symptoms that are a factor inhibiting PA decrease with time after childbirth [4] and physical recovery is expected [26], which suggests that activity may be facilitated. Although a number of studies have examined relationships between parity and PA, no consensus has been obtained [9, 17, 20, 27]. In this study, multiparity showed an association with higher total PA. The latest study using the Pregnancy Physical Activity Questionnaire [27, 28], which includes question items that reflect the lives of women during the child-rearing period, was conducted from December 2019 to September 2021 in Poland [29]. In that study, multiparous women were found to have spent more energy on total PA and household activities, and significantly less on sports and passive rest during the third trimester of pregnancy compared to primiparas. Therefore, multiparous women may engage in higher amounts of PA in association with caring for older children (taking them to and from places, playing in the park, etc.).

In a number of previous studies, childcare support was given as a factor promoting PA, so this study obtained answers for “Number of people who share regular childcare activities” and “Having someone you can talk to with about work and home life?” as childcare support questions. The mean number of persons who shared in childcare duties was 1.3 and 81.2% of respondents had someone with whom they could talk. These women with someone to whom they could talk were found to have a higher total PA than women without such a relationship. Previously, a lack of social supports for childcare was indicated to negatively affect PA and mental health [5, 9, 20]. In a survey on social supports and postpartum depression, the incidence of postpartum depression was 7.5% in a group able to consult with a partner or other person, 36.9% in a group unable to consult with their partner but able to consult with another person, and 63.6% in a group unable to consult with anyone, suggesting the importance of emotional support [30]. Even after one year from April 2020, most delivery facilities in Japan have barred or partially restricted families and other visitors from being present in outpatient clinics, prenatal classes, during delivery, inpatient care, and inpatient visits [31]. Also, under such the circumstances, receiving supports from relatives and people nearby is likely to be much more difficult compared before the pandemic. Meanwhile, mothers with high social support scores even during the COVID-19 epidemic were reported to exhibit better psychological states than mothers with low scores [32]. As mentioned previously, given the relationship between worsening mental state and physical inactivity, against a backdrop of mental health stressors brought on by a pandemic, the existence of someone with whom to consult provides a support to mothers and is also thought to produce positive effects on the PA.

### PA level and HRQoL

The Active group showed higher SF-12v2 scores than the Inactive group. This suggests that mothers with a high PA level have higher HRQoL, supporting a review of 55 papers on the relationship between the PA and QoL, which concluded that a uniform positive relationship exists between PA level and HRQoL [33]. SF-12v2 standard values of Japanese women in their 30 s are as follows: mean PCS, 51.7 (SD 7.7), interquartile range (IQR) 47.1–54.3; mean MCS, 47.4 (SD 9.4), IQR 41.7–48.5. In both groups, PCS was lower than the mean for the general population of the same age and the Inactive group showed a value close to the 25th percentile, suggesting the presence of a certain level of physical discomfort. Moreover, differences in summary score due to activity level were more marked for physical health (3.2 points) than for mental health (1.3 points), representing a similar result to that of a previous study that identified a strong relationship between activity level and physical health. The WHO recommends engaging in moderate PA for ≥ 150 min each week during the postpartum period [34]. In randomized control trials on PA recommended by the WHO, postpartum women who performed continuous PA showed improvements in HRQoL from before to after the interventions [35, 36]. The Active group included women who met one of the following criteria: (1) vigorous PA for ≥ 20 min, ≥ 3 days/week; (2) moderate PA or walking ≥ 30 min, ≥ 5 days/week; or (3) PA on ≥ 5 days/week for the total activity of ≥ 10 MET h/wk. Thus, mothers who are able to perform PA continuously throughout the week appear better able to maintain physical and mental health than mothers who are not.

### Findings to promote postpartum PA

The perception of anxiety under the COVID-19 pandemic appears to contribute to physical inactivity, not only in postpartum women, but also in the general population. Nagata et al. suggested that anxiety about disrupting social harmony by spreading the infection or going out makes Japanese people more inactive [21]. During the survey period, the state of emergency was declared to several prefectures where the infection situation has worsened (1st, April 25 to June 20, 2021; 2nd, July 12 to September 30, 2021). The government focused on requesting, rather than mandating, individuals and businesses to practice social distancing and avoid nonessential activities even under the policy. A feature of Japanese policy is that individuals could make decisions about preventive actions, so different impacts on PA than lockdowns in other countries were predicted. Participants in this study gave birth after March 2020, thus were affected by the pandemic for a long period of time after childbirth. Postpartum women taking care of an infant in such social situations appear likely to experience even more diverse anxieties. The GAD-2 used in screening for anxiety symptoms in this study has been suggested to be related to physical inactivity and may be useful for identifying subjects in need of more support. Continuous examination of factors facilitating PA, with a focus on the variables examined in this study, such as primiparity and women with a shorter postpartum period, would help promote postpartum PA.

## Limitations

Several limitations need to be taken into consideration. First, this cross-sectional study was limited by not having data from before the pregnancy. Second, recruitment was conducted via the Internet, and many participants lived in urban areas, had a long education history and high employment rate, and did not necessarily reflect the socio-demographically average Japanese women. Postpartum PA has been reported to have stronger associations with working status and education [17]. None of these factors were significantly associated with PA in this study. Previous research selected users of designated facilities such as medical institutions or subjects residing in the Tohoku region, and lifestyles may also have differed due to differences in participant characteristics. Third, in terms of measuring PA, since the IPAQ-SF evaluates activities lasting ≥ 10 min, evaluating frequently performed activities (such as housework and childcare) as a PA is difficult because the activity time per activity is short. Therefore, the PA of mothers may have been underestimated compared to results obtainable from an interview format. In fact, according to an American study that evaluated PA in an interview format, total PA (25.7 MET h/wk) for women 3 months after childbirth was higher than that of participants in the present study [7]. Future research should consider the selection bias, and more detailed examinations should be conducted using multifaceted and longitudinal evaluations of PA appropriate to the lifestyle of women in the child-rearing period.

## Conclusions

During the self-refrain situation in Japan, the half of the postpartum women showed inactive levels of PA. Younger age, multiparity, longer duration after childbirth were factors of becoming active lifestyle. The perception of anxiety during the pandemic led negative effects on the PA by the Japanese postpartum women. The presence of someone with whom can talk to about childcare and partner issues was associated with the maintenance of higher PA, suggesting that factor as a positive influence on the PA. Since the impact of mental health and physical inactivity due to COVID-19 pandemic has negative effects on maternal health and QOL, further studies and support focusing on the related factors identified in this study are warranted.

## Data Availability

Due to confidentiality and restrictions being imposed by the research ethics committee of Nippon Sport Science University, the data used in this study have not been deposited in a public repository. The individual responsible for data management is Dr. Yumi Nomura. Data inquiries should be addressed by e-mail (yumi.nmr@gmail.com.).

## Abbreviations

PA: Physical activity
WHO: World Health Organization
COVID-19: Coronavirus disease 2019QoL: quality of life
HRQoL: Health-related quality of life
GAD-2: Two-item generalized anxiety disorder scale
IPAQ-SF: International physical activity questionnaire, short form
MET: Metabolic equivalents
PCS: Physical component summary scale
MCS: Mental component summary scale
SD: Standard deviation
IQR: Interquartile range
OR: Odds ratio
CI: Confidence interval
